# Twenty-one-year report from the Danish Health Authority Expert Advisory Panel for review of treatment of 10 000 cancer patients

**DOI:** 10.1093/oncolo/oyaf059

**Published:** 2025-05-08

**Authors:** Morten Ladekarl, Mette Louise Mørk, Emma Skotte Albertsen, Dorte Nielsen, Ulrik Lassen, Morten Mau-Sørensen, Claus Malta Nielsen, Anders Jakobsen, Hans von der Maase

**Affiliations:** Department of Oncology and Clinical Cancer Research Center, Aalborg University Hospital, 9000 Aalborg, Denmark; Department of Clinical Medicine, Aalborg University, 9220 Aalborg, Denmark; Danish Health Authority, 2300 Copenhagen, Denmark; Danish Health Authority, 2300 Copenhagen, Denmark; Department of Oncology, Copenhagen University Hospital - Herlev and Gentofte, 2730 Herlev, Denmark; Department of Clinical Medicine, Faculty of Health and Medical Sciences, University of Copenhagen, 1172 Copenhagen, Denmark; Department of Clinical Medicine, Faculty of Health and Medical Sciences, University of Copenhagen, 1172 Copenhagen, Denmark; Department of Oncology, Copenhagen University Hospital - Rigshospitalet, 2100 Copenhagen, Denmark; Department of Oncology, Copenhagen University Hospital - Rigshospitalet, 2100 Copenhagen, Denmark; Danish Health Authority, 2300 Copenhagen, Denmark; Department of Oncology, Vejle Hospital, University Hospital of Southern Denmark, 7100 Vejle, Denmark; Department of Regional Health Research, University of Southern Denmark, 5230 Odense, Denmark; Department of Clinical Medicine, Faculty of Health and Medical Sciences, University of Copenhagen, 1172 Copenhagen, Denmark; Department of Oncology, Copenhagen University Hospital - Rigshospitalet, 2100 Copenhagen, Denmark

**Keywords:** second opinion of treatment, drug reimbursement, experimental treatment, regulatory drug approval, equal drug access, clinical trial units

## Abstract

**Background:**

Patients with hard-to-treat or rare cancers and those not responding to standard-of-care (SoC) treatment have unmet needs. Limited access to novel drugs is an increasing additional challenge. In 2003, the Danish government adopted a Health Act to ensure that treatment of patients with life-threatening disease could be reevaluated by independent experts. The Danish Health Authority (DHA) set up an Expert Advisory Panel to provide advice on possibilities for further treatment of patients, including treatment not approved nationally. A few years later, clinical units were established that could offer unestablished treatment to patients by referral from the Panel. The treatment was first reimbursed by the Government and later by regional authorities.

**Materials and methods:**

We present the structure, workflow, and impact of the Health Act for 21 years for patients with cancer. Annual reports from the DHA were the primary data source.

**Results:**

11 034 cases from 9603 cancer patients were evaluated by the Panel from 2003 to 2023, representing a median of 372 unique cases yearly. In 53%, the Panel advised on further treatment in Denmark, and of these, 56% were recommended nationally nonapproved treatment, 21% SoC treatment or workup, and 19% clinical trial participation. In 4.5% of cases, advice was given on treatment abroad. A significant decline in admissions to the Panel from a peak of 1167 patients in 2008 to 3-400 yearly from 2012 to 2017 followed the conversion of nonapproved treatments to SoC practice. A shift in drug reimbursement, independent of Panel advise, reduced the clinical impact and explained the further decline observed in admissions lately to only 51 patients in 2023.

**Conclusions:**

This unique national scheme provided early access to treatment for patients with no further SoC options and facilitated the introduction of new cancer treatments, initiation of clinical trials, and establishment of trial units in the country. The scheme may be adapted to other countries with a public healthcare system. Results of the current report indicate that impact is dependent on delivering clinical units and reimbursement associated with the recommended treatment.

**Implications for Practice**

Unmet medical needs in patients with resistant tumors and limited access to novel drugs are frequent challenges in cancer healthcare. Following a Governmental Health Act, the Danish Health Authority set up an Expert Panel to advice on treatment of patients with life-threatening diseases and no further standard-of-care (SoC) possibilities. A total of 11 034 cases from 9603 cancer patients were evaluated from 2003 to 2023. In 53% of cases, the Expert Panel advised on further treatment in Denmark. Of these, 56% were recommended nationally nonapproved treatment, 21% SoC treatment, and 19% trial participation. In 4.5%, advice was given on treatment abroad. This unique initiative provided early access to novel drugs and facilitated initiation of clinical trials and establishment of trial units. Results indicate that impact is dependent on delivering clinical facilities and associated reimbursement of recommended treatment.

## Introduction

Although most patients with incurable cancer can be offered evidence-based treatment throughout their course of disease, medical needs are unmet in hard-to-treat or rare cancers and in patients not responding to standard treatment.^1^ Restricted access to novel drugs is an additional challenge, with a growing number of proven efficient but expensive pharmaceuticals being marketed but lacking national reimbursement or implementation.^2^

In Denmark, oncological treatment is provided by public hospitals and is free of charge for patients. Introduction of new antineoplastic drugs has historically been driven by oncologists, and reimbursement of expensive treatment regimens was decided by governmental committees including medical experts.^3^ Since 2017, the approval of new drugs in Denmark has been regulated by the Danish Medicines Council (DMC), run by the hospital administrators in 5 Danish Regions.^4,5^ Applications can solely by filed by the industry. The DMC reviews available data and negotiates the price of treatment based on an assessment of cost-benefit according to the method of Quality Adjusted Life Years (QUALY).^5,6^ Although all cancer drugs evaluated by DMC was already approved by the European Medicines Agency (EMA), in a recent status, 40% of applications were not grated reimbursement.^7^ Moreover, the time to decision was on average more than 20 weeks.^7^ Several EMA-approved drugs were not evaluated as the producer did not apply or subsequently withdrew submitted applications.^8^

In 2003, the Danish government adopted a Health Act (Supplementary Appendix) to secure that the treatment of patients with life-threatening disease could be reevaluated by independent experts. As a result, the Danish Health Authority (DHA) set up an Expert Advisory Panel, in the following denoted “the Panel.” The Panel should provide advice about the possibilities of treatment for individuals with life-threatening disease, including treatment not approved nationally. An Executive Order issued in 2017 and updated in 2019 (Supplementary Appendix), limited the Health Act to include only patients who had exhausted their standard-of-care (SoC) options. The same year, a consensus paper on the prioritization of the use of medicines adopted by all parties in the Parliament ensured equal access to drugs, including those rejected by DMC as SoC, provided that the treatment was justified by a specific assessment on an individual basis (Supplementary Appendix). Whether this consensus agreement has worked in practice is currently subject to debate.^9^

Here, we present the infrastructure and resulting effects of the Danish Governmental Health Act for expert review over a period of more than 2 decades. As cases referred to the Panel almost exclusively concerned medical treatment of patients with malignant solid tumors, the few nonmalignant cases (1%), surgical or hematological cases, were not included in this report.

## Methods

### Patient referral

As determined by the Health Act, the treating physician can seek advice by the Panel if the patient suffers from a life-threatening disease. The clinical case is submitted to DHA with a complete presentation of the medical history, including relevant extracts of the medical record such as radiological, pathological, molecular, and laboratory records. The patients are informed by a personal letter from DHA confirming the referral, and the DHA assesses whether referrals are complete and fall within the scope of the Executive Order of the Health Act before the request for advice is sent to the Panel.

### The Expert Advisory Panel

The permanent members of the Panel are clinicians with comprehensive research and expertise in treating cancer. The Panel had for the first 13 years only 2 permanent members but was later expanded to up to 4 clinical oncologists, and up to 3 hematologists and 2 surgeons. In addition, the DHA may appoint ad hoc members with specific knowledge to evaluate rare cases. Permanent members are recruited nationwide and are currently appointed by the DHA for a period of 2 years that may be extended. The members must yearly present a satisfactory Conflicts of Interest (COI) declaration that is published on the home page of DHA.^10^

### Units for experimental oncological treatment

Clinical units—named “Experimental Units”—were established in 2005 at 6 major oncological Departments as a large number of patients were recommended treatment that could not be adapted within the existing frames. Together the Units established early access programs and clinical trials of relevance for patients evaluated by the Panel. One of the units evolved to become a dedicated phase 1 trial unit, located at Rigshospitalet, Copenhagen.^11^

### Government

**Figure 1.**
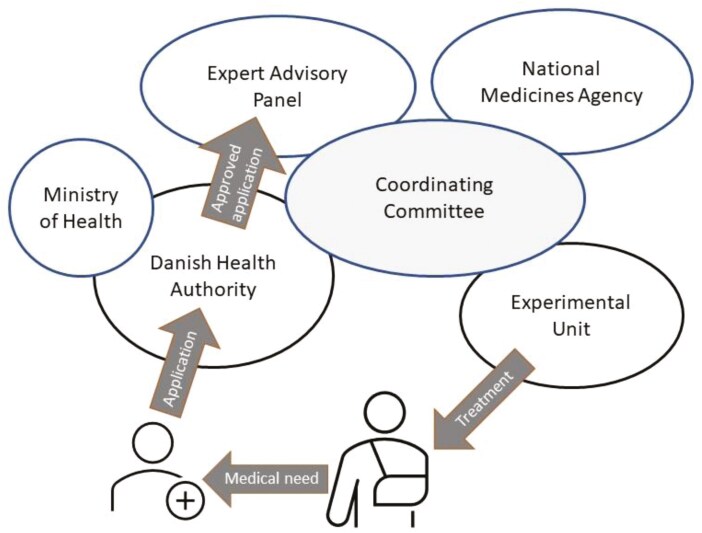
The scheme for Expert Advisory Panel review and treatment in Experimental Units. The application for review of a patient’s case is prepared by the treating physician and sent to the Danish Health Authority who reviews the application according to the Executive Order of the Governmental Health Act. If approved, the application is forwarded to the Expert Advisory Panel for assessment. Emergent medical needs, identified by the Panel based on cases reviewed, are discussed in the Coordinating Committee, and by agreement, one or more of 6 Experimental Units establish the treatment.

The organization of the infrastructure as it was from 2005 is illustrated in Figure 1. Based on referrals, treatment-emergent medical needs were identified by the Panel and discussed in an Executive Committee. The Committee was headed by DHA with participation of all members of the Panel, the Danish Medicines Agency (DMA), and Heads of the Experimental Units. If the Executive Committee agreed upon an unmet medical need, the Experimental Units could immediately establish new treatments with an optimal geographical distribution. Patient referral to the Experimental Units required either prior review and approval of individual cases by the Panel or fulfillment of eligibility criteria specified by the Panel. Treatment in the Units was reimbursed, and administrative tasks were supported by a grant per patient, initially provided by the government and, from 2007, by Danish Regions.

### Panel decisions

For the case assessment, the Panel includes all available scientific information such as published clinical studies, reports from health authorities including European Medicines Agency (EMA) and US Food and Drug Administration (FDA), lectures and abstracts from scientific conferences, trials in progress, and clinical experience. If deemed necessary, the Panel obtains additional advice from national or international experts. One of the Panel members makes a preliminary evaluation which is reviewed by at least one other member. In difficult cases or in cases with discrepant opinion, a consensus is sought among all members.

On a case-by-case basis, the Panel assesses whether available evidence is sufficient to recommend a specific treatment for the patient and whether the expected effect of treatment outweighs risks and potential side effects. The Panel can advise on any treatment modality including but not limited to medical treatment, surgery, or radiotherapy. Treatment abroad may be suggested in the absence of relevant treatment in Denmark. Decisions are categorized prospectively by members of the Panel.

The Panels’ response is submitted to the treating Department who has the final authority to decide whether to follow the advice and is responsible for seeking reimbursement. Furthermore, the attending physician assesses the patients’ current clinical condition and comorbidities and decides whether the patient is eligible. Similarly, based on shared decision, weighting information about benefit and risks, the patient can assess whether he/she consents to the proposed treatment.

### Data source

The primary data source of this report is annual reports published online by DHA (Supplementary Appendix). Reports are based on prospectively registered information on patients’ characteristics, administrative data, and panel advice. A copy of the database used is provided in Supplementary database.

### Ethics

This study is based on publicly available reports. No permission from authorities or patients is required for such studies in Denmark.

## Results

### Case characteristics

**Figure 2.**
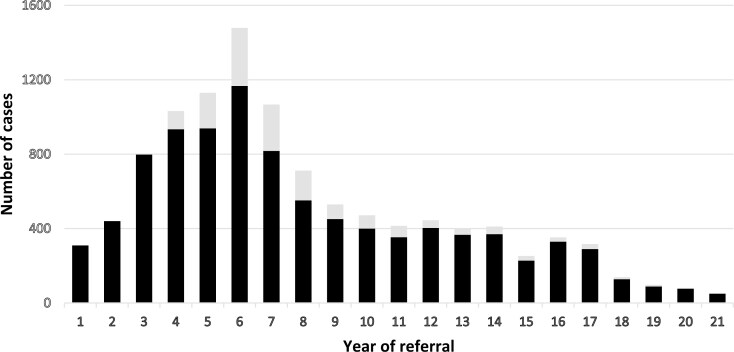
The number of new patient cases assessed by the Expert Advisory Panel (black bars), and the number of cases reassessed (gray bars) per year (2003-2023). Note: Number of reassessed cases not registered years 1-3.

A total of 11 034 cases were evaluated by the Panel in a 21-year period from 2003 to the end of 2023. Of these 1431 (13%) were reassessments and 9603 were unique cases. Patients referred had a median age of 59 years (range, 1-90 years), equally distributed across genders. Per year, the median number of unique cases evaluated was 372. Figure 2 shows a rapid increase in annual cases from 2003 to a peak in 2008 where 1167 unique cases were evaluated. The number of cases declined thereafter to a plateau of 3-400 cases in 2012-2017 followed by a further decrease to a minimum of 51 cases in 2023. Registered from 2017 and onward, an additional 241 cases were referred but rejected by the DHA, representing 17% of cases these years. The reason for administrative rejection was requests for evaluation of treatment already established as SoC in the majority of cases (Supplementary Table S1). In total, 89% of cases were referred by physicians used at oncological departments. The distribution of primary tumor types of patients referred varied with year of referral, reflecting diagnosis-dependent changing treatment opportunities (Supplementary Figure S1).

### Panel advises and treatment

**Figure 3.**
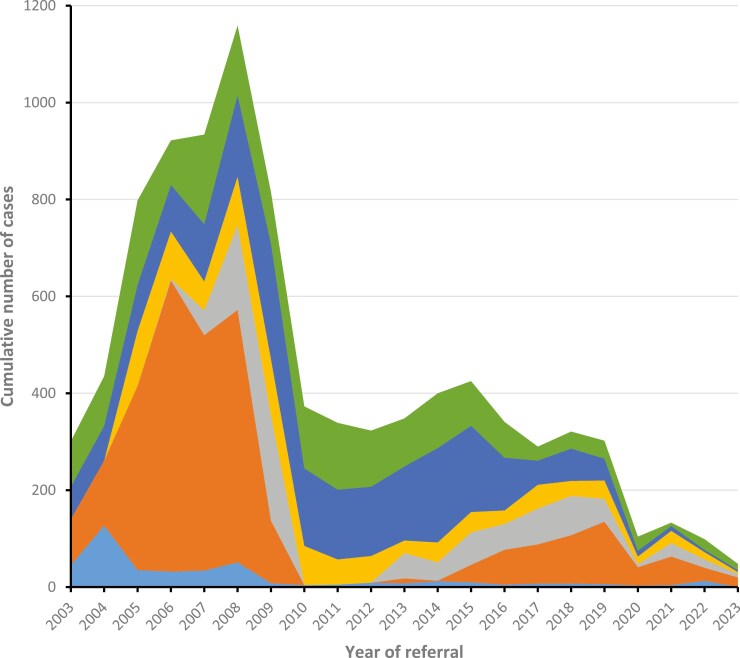
Cumulative numbers of primary advice given by the Expert Advisory Panel per year of referral, distributed according to advice categories. Color codes according to type of primary advise: light blue, treatment abroad; orange, nonreimbursed treatment; dark blue, suggested treatment endorsed; green, no further suggestions; yellow, further standard-of-care treatment, further investigations, or second opinion of treatment suggested; and gray, advise on treatment in a clinical trial.

Figure 3 illustrates the number of primary Panel advises distributed according to categories per year of referral. The relative distribution is shown in Supplementary Figure S2. In the early years, a clear majority of advises recommended nonreimbursed treatments with a peak of 601 cases in 2006. In 2005, Experimental Units were established and 370 patients were referred to the 6 Units this year. The numbers treated in the Experimental Units peaked in 2008 with 693 patients but declined to 107 in 2011. With the initiation of a range of clinical trials and early access programs conducted at the Experimental Units and increased national approval of novel drugs, the numbers of advice of nonreimbursed treatments declined drastically concurrently with an increase in advices endorsing treatments suggested by the referring physician and suggestions on further SoC treatment or work up. In the same period, advice on treatments delivered abroad decreased substantially and remained a rare advice throughout the rest of the period.

In the 21-year period, the majority of patients (53%) were advised on further treatment in Denmark. Of these, 56% were recommended nationally nonapproved treatment, 21% SoC treatment or work up, and 19% trial participation. In a total of 42% of cases, no further suggestions on treatment were provided. The entity could be divided into an equal number of cases for whom the Panel endorsed the treatment suggested by the referring physician, and cases where no further treatment could be suggested.

A total of 425 (4.5%) patients were advised on treatment abroad. Of these, 60% were advised on treatment with drugs that were not reimbursed in Denmark, and 40% were advised on clinical trial participation. Treatment abroad was provided in European countries, Japan, and the USA. Seven patients were advised on SoC treatment not available in Denmark from 2007 to 2011. Thereafter, no cases were evaluated by the Panel as this kind of treatment was then approved directly by the DHA.

### Turnaround time

The average turnaround time from application received to Panel answer was returned was 11 days (range in yearly average, 7-18 days). On average, 80% of answers were returned within 2 weeks (yearly range, 62%-92%). The processing time was extended mainly if supplementary medical information was required or if Panel specialists of more than 1 specialty or additional expert knowledge outside the Panel was needed (data not shown).

## Discussion

To our knowledge, this governmental initiative offering centralized expert committee-advice on treatment of individual patients is unique to Denmark. It has inspired a similar initiative in Norway beginning in 2022^12^ and may be adapted to other countries with a public healthcare system. Although this 21-year report demonstrates the continuous use of the scheme, with ~ 15 500 people dying from cancer yearly in the nation,^13^ the fraction of cases referred to the Panel is small, ranging from 7.5% at its peak in 2008 to 0.3% in 2023. The recent drastic fall in use seems paradoxical and potential reasons for variation over time in the referral pattern are discussed below.

Although the purpose of the governmental Health Act is to review cases on an individual basis, the initiative has historically had an important impact on oncology in Denmark in general. In particular, the establishment of geographically widespread Experimental Units with new treatments being suggested by the Executive Committee and patients’ eligibility being determined by the Panel, immediately resulted in an accelerated access to novel anticancer drugs. From the patients’ perspective, the initiative had important implications, as those referred to treatment at the Experimental Units had no further standard treatment options at that time.

For example, patients with metastatic renal cell carcinoma (RCC) could be treated with tyrosine-kinase inhibitors (TKI’s) from 2006, although the first positive phase III-results were published in January 2007.^14,15^ In a review of 1073 RCC-patients treated at the Experimental Units, it was shown that introduction of TKI’s was accompanied by an improved survival of the diseased population compared with historical controls.^16^ Sorafenib and sunitinib for RCC were established as SoC in Denmark in 2009. Similarly, following trial results presented at the Annual Meeting of the American Society of Clinical Oncology in June 2007,^17^ patients with advanced hepatocellular carcinoma (HCC) could be offered sorafenib from August the same year. A retrospective assessment of all 76 cases treated at the Experimental Units until 2009 showed poor survival outcome of HCC patients with impaired liver function or poor PS.^18^ The treatment was in 2009 implemented as SoC in Denmark and recommended in the national guideline only for fit patients. In July 2005, positive results of erlotinib targeting EGFR in unselected patients with non-small cell lung cancer (NSCLC) were published.^19^ Erlotinib was first offered to Danish patients selected by clinical and histological characteristics by referral to centers abroad and later to the Experimental Units in Denmark. In a cohort study of 488 Danish patients, molecular reassessment showed efficacy of the treatment confined to patients with tumors harboring EGFR mutations,^20^ supporting international data.^21^ Erlotinib was established as SoC in Denmark in 2007 for patients with EGFR-mutated NSCLC. In 2007-2008, most patients recommended treatment abroad were referred to Basel, Switzerland, for treatment of neuroendocrine tumors with peptide receptor radiation therapy.^22^ A retrospective review of the 69 patients treated showed an objective response rate of 24% and stable disease in 62%,^23^ and from 2008, PPRT was introduced in Denmark at 2 centers. Proof of survival benefit was, however, first established 9 years later.^24^

The initiative promoted early access to an additional range of emerging novel treatments (year of published pivotal data in parenthesis) including imatinib for gastrointestinal stromal tumor (GIST) (2002),^25^ platinum and pemetrexed for mesothelioma (2003),^26^ docetaxel for castration-resistant prostatic cancer (2004),^27^ adjuvant platinum and vinorelbine for NSCLC (2005),^28^ sunitinib for GIST (2006),^29^ cetuximab for colorectal cancer (2007),^30^ everolimus^31^ and sunitinib^32^ for pancreatic neuroendocrine tumors (2011), pazopanib for sarcomas (2012),^33^ taxanes for platinum-resistant gastro-esophageal cancer (2012),^34,35^ nilotinib for GIST (2012),^36^ regorafenib for GIST^37^ and colorectal cancer^38^ (2013), and trabectedin for sarcomas (2016).^39^ Finally, early access to ipilimumab was provided by the Experimental Units anticipating the revolution of immune check point inhibitors for treatment of malignant melanoma (2011).^40^ Common to most of these treatments, they were judged to provide benefit to patients by FDA and EMA and were later approved as SoC by Danish authorities. However, in an assessment of the clinical benefit of solid tumor drugs in 135 noncurative indications that were EMA-approved between January 2009 and October 2020, only 28.9% and 15.6% met the Magnitude of Clinical Benefit (MCB) criteria of the original and adapted ESMO-MCBS framework (scores 4-5), respectively, indicating substantial clinical benefit.^41^

The initiative also facilitated the first certified phase 1 trial unit in Denmark^11^ and boosted the initiation of investigator-initiated clinical trials established to treat patients with medical needs as determined by the Executive Committee. These included Panel-endorsed trials of stereotactic body radiotherapy^42^ and phase I- and II-studies of first-line treatment of biliary tract carcinomas,^43,44^ platinum-resistant ovarian cancer,^45^ chemo-refractory upper gastrointestinal cancers,^46^ third-line treatment of colorectal cancer,^47^ second-line treatment of pancreatic cancer (NCT01042028) and gliomas,^48^ and hepatic intra-arterial chemotherapy for liver metastases.^49,50^ Most of these trials contributed with significant knowledge to the oncologic community, but none proceeded to phase III.

A significant decline in admissions to the Panel from a peak in 2008 followed the national approval of many new treatments as well as some were introduced in earlier lines of therapy.^51^ The further drop in referrals observed from 2020 coincides with a shift toward a more restricted practice in drug reimbursement. The rapid increase in drug expenses^52^ has led to restrictions to reimburse the use of EMA-approved drugs, including several with high-level evidence of benefit.^53,54^ Thus, due to the lack of reimbursement, more patients may no longer be offered the treatment suggested by the Panel, limiting the role of the Health Act. A new challenge has evolved through increased use of molecular testing of cancer patients in search of predictive markers for precision medicine.^55,56^ Drugs for targeted treatment are often based on low level of evidence due to rarity of specific variants^57^ and are often not reimbursed when approval is based on QUALY.^58^ Hence, while unregistered targeted treatment of unproven efficacy may be available to patients in clinical trials,^59^ patients who harbor targets druggable by EMA- or FDA-approved drugs may not be offered treatment. Similar paradoxes are recognized in other healthcare systems,^60^ and alternative solutions to secure treatment access for precision medicine are urgently needed.^61^

The strength of this study is the reporting on a unique governmental initiative of early access to treatment by centralized expert case review that may inspire similar initiatives. Due to regulatory restrictions, we lack data on whether advises were followed as well as data on outcome of suggested treatments, including compliance, efficacy, and toxicity. However, studies of specific cohorts have been published as referenced above. As the MCB of most newly approved cancer therapies is low even in patients selected for clinical trial participation,^41^ real-world data on efficacy and tolerability of novel drugs in unselected populations are highly warranted.^62^

In conclusion, we present the structure and prospectively collected data from 21 years of a national governmental initiative offering centralized expert-panel review of individual cases of cancer patients with exhausted SoC and reimbursed treatment options. More than 10 000 cases were evaluated by the Expert Panel, and in 53% of these, advise for additional treatment was given, including advise on early access to nonreimbursed drugs. Historically, the initiative contributed to the uncovering of medical needs, facilitated introduction of novel anti-cancer regimens, and contributed to the establishment of phase I trial units and investigator-driven clinical trials. While the usefulness of the scheme is today challenged by altered governance of reimbursement, in some aspect’s history repeats as access to modern drugs is increasingly restricted. Searching for solutions to current issues of accessibility of expensive drugs to treat cancer patients, a revitalization of the described scheme could be considered. The present report indicates that impact is highly dependent on delivering clinical units providing novel treatments and a close connection between medical advice and reimbursement as well as systematic follow-up of results should be mandatory in future initiatives.

## Supplementary Material

oyaf059_suppl_Supplementary_Appendix

oyaf059_suppl_Supplementary_Datatabase

oyaf059_suppl_Supplementary_Tables_S1_Figures_S1-S2

## Data Availability

The data underlying this article are available in the article and in its online supplementary material.

## References

[1] Barrenho E , Halmai R, Miraldo M, et al Inequities in cancer drug development in terms of unmet medical need. Soc Sci Med. 2022;302:114953. https://doi.org/10.1016/j.socscimed.2022.114953 35489114

[2] OECD. *Addressing Challenges in Access to Oncology Medicines*. https://www.oecd.org/health/health-systems/addressing-challenges-in-access-to-oncology-medicines.htm

[3] RADS. *The Danish Council for the Use of Expensive Hospital Medicines*, 2009. https://www.regioner.dk/media/2830/radsfolder-engelsk.pdf

[4] Danish Medicines Council. *Danish Medicines Council*, 2024. https://medicinraadet.dk/om-os/in-english

[5] AMGROS. *New Pharmaceuticals and Negotiations*, 2024. https://amgros.dk/en/pharmaceuticals/price-negotiations-and-tendering/new-pharmaceuticals-and-negotiations/

[6] Devlin NJ , Lorgelly PK. QALYs as a measure of value in cancer. J Cancer Policy. 2017;11:19-25. https://doi.org/10.1016/j.jcpo.2016.09.005

[7] Medicinrådet. *Kortere Sagsbehandlingstider*, 2024. https://medicinraadet.dk/nyheder/2024/kortere-sagsbehandlingstider

[8] EMA. *National Registers of Authorised Medicines*, 2024. https://www.ema.europa.eu/en/medicines/national-registers-authorised-medicines

[9] Alfthan C. Patientformand: Det 7. princip fungerer ikke som et sikkerhedsnet. *Sundhedspolitisk Tidsskr*, 2024. https://sundhedspolitisktidsskrift.dk/nyheder/sundhedspolitik/8637-patientformand-det-7-princip-loser-ikke-problemet.html

[10] Danish Health Authority. *Councils, Committees and Advisers*, 2024. Accessed December 8, 2024. https://www.sst.dk/en/english/About-us/Organisation/Councils-committees-and-advisers

[11] Rigshospitalet. Phase 1 Unit, 2024. Accessed December 8, 2024. https://www.rigshospitalet.dk/english/research-and-innovation/units-and-groups/phase-1-unit/Pages/default.aspx

[12] Helse Norge. *Right to Re-assessment*, 2024. Accessed December 8, 2024. https://www.helsenorge.no/en/health-rights-in-norway/sykehus-og-spesialist/rett-til-fornyet-vurdering/#when-can-you-ask-for-your-referral-to-be-reassessed

[13] Statistica. *Number of Cancer Deaths in Denmark from 2008 to 2021, by Gender*, 2024. https://www.statista.com/statistics/968333/number-of-deaths-due-to-cancer-in-denmark-by-gender/

[14] Escudier B , Eisen T, Stadler WM, et al Sorafenib in advanced clear-cell renal-cell carcinoma. N Engl J Med. 2007;356:125-134. https://doi.org/10.1056/NEJMoa060655 17215530

[15] Motzer RJ , Hutson TE, Tomczak P, et al Sunitinib versus interferon alfa in metastatic renal-cell carcinoma. N Engl J Med. 2007;356:115-124. https://doi.org/10.1056/NEJMoa065044 17215529

[16] Soerensen AV , Donskov F, Hermann GG, et al Improved overall survival after implementation of targeted therapy for patients with metastatic renal cell carcinoma: results from the Danish Renal Cancer Group (DARENCA) study-2. Eur J Cancer. 2014;50:553-562. https://doi.org/10.1016/j.ejca.2013.10.010 24215846

[17] Llovet J , Ricci S, Mazzaferro V, et al Randomized phase III trial of sorafenib versus placebo in patients with advanced hepatocellular carcinoma (HCC). J Clin Oncol. 2007;25:LBA1-LBA1. https://doi.org/10.1200/jco.2007.25.18_suppl.lba1

[18] Køstner AH , Sorensen M, Olesen RK, et al Sorafenib in advanced hepatocellular carcinoma: a nationwide retrospective study of efficacy and tolerability. Sci World J. 2013;2013:1-6. https://doi.org/10.1155/2013/931972 PMC3569916 23431262

[19] Shepherd FA , Rodrigues Pereira J, Ciuleanu T, et al Erlotinib in previously treated non–small-cell lung cancer. N Engl J Med. 2005;353:123-132. https://doi.org/10.1056/NEJMoa050753 16014882

[20] Weber B , Hager H, Sorensen BS, et al EGFR mutation frequency and effectiveness of erlotinib: a prospective observational study in Danish patients with non-small cell lung cancer. Lung Cancer. 2014;83:224-230. https://doi.org/10.1016/j.lungcan.2013.11.023 24388704

[21] da Cunha Santos G , Shepherd FA, Tsao MS. EGFR mutations and lung cancer. Annu Rev Pathol. 2011;6:49-69. https://doi.org/10.1146/annurev-pathol-011110-130206 20887192

[22] Forrer F , Waldherr C, Maecke HR, Mueller-Brand J. Targeted radionuclide therapy with 90Y-DOTATOC in patients with neuroendocrine tumors. Anticancer Res. 2006;26:703-707. http://www.ncbi.nlm.nih.gov/pubmed/16739341 16739341

[23] Pfeifer AK , Gregersen T, Grønbæk H, et al Peptide receptor radionuclide therapy with 90Y-DOTATOC and 177Lu-DOTATOC in advanced neuroendocrine tumors: results from a Danish cohort treated in Switzerland. Neuroendocrinology. 2011;93:189-196. https://doi.org/10.1159/000324096 21335949

[24] Strosberg J , El-Haddad G, Wolin E, et al Phase III trial of 177 Lu-Dotatate for midgut neuroendocrine tumors. N Engl J Med. 2017;376:125-135. https://doi.org/10.1056/NEJMoa1607427 28076709 PMC5895095

[25] Demetri GD , von Mehren M, Blanke CD, et al Efficacy and safety of imatinib mesylate in advanced gastrointestinal stromal tumors. N Engl J Med. 2002;347:472-480. https://doi.org/10.1056/NEJMoa020461 12181401

[26] Vogelzang NJ , Rusthoven JJ, Symanowski J, et al Phase III study of pemetrexed in combination with cisplatin versus cisplatin alone in patients with malignant pleural mesothelioma. J Clin Oncol. 2003;21:2636-2644. https://doi.org/10.1200/JCO.2003.11.136 12860938

[27] Tannock IF , de Wit R, Berry WR, et al Docetaxel plus prednisone or mitoxantrone plus prednisone for advanced prostate cancer. N Engl J Med. 2004;351:1502-1512. https://doi.org/10.1056/NEJMoa040720 15470213

[28] Winton T , Livingston R, Johnson D, et al Vinorelbine plus cisplatin vs. observation in resected non–small-cell lung cancer. N Engl J Med. 2005;352:2589-2597. https://doi.org/10.1056/NEJMoa043623 15972865

[29] Demetri GD , van Oosterom AT, Garrett CR, et al Efficacy and safety of sunitinib in patients with advanced gastrointestinal stromal tumour after failure of imatinib: a randomised controlled trial. Lancet. 2006;368:1329-1338. https://doi.org/10.1016/S0140-6736(06)69446-4 17046465

[30] Jonker DJ , O’Callaghan CJ, Karapetis CS, et al Cetuximab for the treatment of colorectal cancer. N Engl J Med. 2007;357:2040-2048. https://doi.org/10.1056/NEJMoa071834 18003960

[31] Yao JC , Shah MH, Ito T, et al Everolimus for advanced pancreatic neuroendocrine tumors. N Engl J Med. 2011;364:514-523. https://doi.org/10.1056/NEJMoa1009290 21306238 PMC4208619

[32] Raymond E , Dahan L, Raoul JL, et al Sunitinib malate for the treatment of pancreatic neuroendocrine tumors. N Engl J Med. 2011;364:501-513. https://doi.org/10.1056/NEJMoa1003825 21306237

[33] van der Graaf WT , Blay JY, Chawla SP, et al Pazopanib for metastatic soft-tissue sarcoma (PALETTE): a randomised, double-blind, placebo-controlled phase III trial. Lancet. 2012;379:1879-1886. https://doi.org/10.1016/S0140-6736(12)60651-5 22595799

[34] Ford HER , Marshall A, Bridgewater JA, et al Docetaxel versus active symptom control for refractory oesophagogastric adenocarcinoma (COUGAR-02): an open-label, phase III randomised controlled trial. Lancet Oncol. 2014;15:78-86. https://doi.org/10.1016/S1470-2045(13)70549-7 24332238

[35] Ueda S , Hironaka S, Yasui H, et al Randomized phase III study of irinotecan (CPT-11) versus weekly paclitaxel (wPTX) for advanced gastric cancer (AGC) refractory to combination chemotherapy (CT) of fluoropyrimidine plus platinum (FP): WJOG4007 trial. J Clin Oncol. 2012;30:4002-4002. https://doi.org/10.1200/jco.2012.30.15_suppl.4002

[36] Reichardt P , Blay JY, Gelderblom H, et al Phase III study of nilotinib versus best supportive care with or without a TKI in patients with gastrointestinal stromal tumors resistant to or intolerant of imatinib and sunitinib. Ann Oncol. 2012;23:1680-1687. https://doi.org/10.1093/annonc/mdr598 22357255

[37] Demetri GD , Reichardt P, Kang YK, et al Efficacy and safety of regorafenib for advanced gastrointestinal stromal tumours after failure of imatinib and sunitinib (GRID): an international, multicentre, randomised, placebo-controlled, phase III trial. Lancet. 2013;381:295-302. https://doi.org/10.1016/S0140-6736(12)61857-1 23177515 PMC3819942

[38] Grothey A , Cutsem EV, Sobrero A, et al Regorafenib monotherapy for previously treated metastatic colorectal cancer (CORRECT): an international, multicentre, randomised, placebo-controlled, phase III trial. Lancet. 2013;381:303-312. https://doi.org/10.1016/s0140-6736(12)61900-x 23177514

[39] Demetri GD , von Mehren M, Jones RL, et al Efficacy and safety of trabectedin or dacarbazine for metastatic liposarcoma or leiomyosarcoma after failure of conventional chemotherapy: results of a phase III randomized multicenter clinical trial. J Clin Oncol. 2016;34:786-793. https://doi.org/10.1200/JCO.2015.62.4734 26371143 PMC5070559

[40] Robert C , Thomas L, Bondarenko I, et al ipilimumab plus dacarbazine for previously untreated metastatic melanoma. N Engl J Med. 2011;364:2517-2526. https://doi.org/10.1056/NEJMoa1104621 21639810

[41] Grössmann N , Wolf S, Rothschedl E, Wild C. 12 years of European cancer drug approval—a systematic investigation of the ‘magnitude of clinical benefit.’. ESMO Open. 2021;6:100166. https://doi.org/10.1016/j.esmoop.2021.100166 34087744 PMC8182388

[42] Høyer M , Muren LP. Stereotactic body radiation therapy—a discipline with Nordic origin and profile. Acta Oncol. 2012;51:564-567. https://doi.org/10.3109/0284186x.2012.684869 22574782

[43] Lassen U , Jensen LH, Sorensen M, et al A Phase I–II dose escalation study of fixed-dose rate gemcitabine, oxaliplatin and capecitabine every 2 weeks in advanced cholangiocarcinomas. Acta Oncol. 2011;50:448-454. https://doi.org/10.3109/0284186x.2010.500300 20670085

[44] Amin NEL , Hansen TF, Fernebro E, et al Randomized Phase II trial of combination chemotherapy with panitumumab or bevacizumab for patients with inoperable biliary tract cancer without KRAS exon 2 mutations. Int J Cancer. 2021;149:119-126. https://doi.org/10.1002/ijc.33509 33561312

[45] Boisen MK , Madsen CV, Dehlendorff C, et al The prognostic value of plasma YKL-40 in patients with chemotherapy-resistant ovarian cancer treated with bevacizumab. Int J Gynecol Cancer. 2016;26:1390-1398. https://doi.org/10.1097/IGC.0000000000000798 27648712

[46] Rohrberg KS , Olesen RK, Pfeiffer P, et al Phase II trial of erlotinib and bevacizumab in patients with advanced upper gastrointestinal cancers. Acta Oncol. 2012;51:234-242. https://doi.org/10.3109/0284186X.2011.619568 22017239

[47] Pfeiffer P , Nielsen D, Bjerregaard J, et al Biweekly cetuximab and irinotecan as third-line therapy in patients with advanced colorectal cancer after failure to irinotecan, oxaliplatin and 5-fluorouracil. Ann Oncol. 2008;19:1141-1145. https://doi.org/10.1093/annonc/mdn020 18281264

[48] Møller S , Grunnet K, Hansen S, et al A phase II trial with bevacizumab and irinotecan for patients with primary brain tumors and progression after standard therapy. Acta Oncol. 2012;51:797-804. https://doi.org/10.3109/0284186X.2012.681063 22548369

[49] Nielsen DL , Nørgaard H, Vestermark LW, et al Intrahepatic and systemic therapy with oxaliplatin combined with capecitabine in patients with hepatic metastases from breast cancer. Breast. 2012;21(4):556-561 https://doi.org/10.1016/j.breast.2012.05.003 22672848

[50] Abrahamsson H , Jensen BV, Berven LL, et al Antitumour immunity invoked by hepatic arterial infusion of first‐line oxaliplatin predicts durable colorectal cancer control after liver metastasis ablation: 8–12 years of follow‐up. Int J Cancer. 2020;146:2019-2026. https://doi.org/10.1002/ijc.32847 31872440

[51] Michaeli DT , Michaeli JC, Michaeli T. Advances in cancer therapy: clinical benefit of new cancer drugs. Aging. 2023;15(12):5232. https://doi.org/10.18632/aging.204839 37338507 PMC10333065

[52] Torkki P , Leskelä RL, Bugge C, Torfadottir JE, Karjalainen S. Cancer-related costs should be allocated in a comparable way—benchmarking costs of cancer in Nordic countries 2012-2017. Acta Oncol. 2022;61:1216-1222. https://doi.org/10.1080/0284186X.2022.2124883 36151990

[53] Modi S , Jacot W, Yamashita T, et al Trastuzumab deruxtecan in previously treated HER2-low advanced breast cancer. N Engl J Med. 2022;387:9-20. https://doi.org/10.1056/NEJMoa2203690 35665782 PMC10561652

[54] Powles T , Rosenberg JE, Sonpavde GP, et al Enfortumab vedotin in previously treated advanced urothelial carcinoma. N Engl J Med. 2021;384:1125-1135. https://doi.org/10.1056/NEJMoa2035807 33577729 PMC8450892

[55] Nassar SF , Raddassi K, Ubhi B, Doktorski J, Abulaban A. Precision medicine: steps along the road to combat human cancer. Cells. 2020;9:2056. https://doi.org/10.3390/cells9092056 32916938 PMC7563722

[56] Ladekarl M , Nøhr AK, Sønderkær M, et al Feasibility and early clinical impact of precision medicine for late-stage cancer patients in a regional public academic hospital. Acta Oncol. 2023;62:261-271. https://doi.org/10.1080/0284186x.2023.2185542 36905645

[57] Mateo J , Chakravarty D, Dienstmann R, et al A framework to rank genomic alterations as targets for cancer precision medicine: the ESMO Scale for Clinical Actionability of molecular Targets (ESCAT). Ann Oncol. 2018;29:1895-1902. https://doi.org/10.1093/annonc/mdy263 30137196 PMC6158764

[58] Weymann D , Pollard S, Lam H, Krebs E, Regier DA. Toward best practices for economic evaluations of tumor-agnostic therapies: a review of current barriers and solutions. Value Health. 2023;26:1608-1617. https://doi.org/10.1016/j.jval.2023.07.004 37543205

[59] Kringelbach T , Højgaard M, Rohrberg K, et al ProTarget: a Danish Nationwide Clinical Trial on Targeted Cancer Treatment based on genomic profiling—a national, phase II, prospective, multi-drug, non-randomized, open-label basket trial. BMC Cancer. 2023;23:182. https://doi.org/10.1186/s12885-023-10632-9 36814246 PMC9948467

[60] Gordon N , Goldstein DA, Tadmor B, Stemmer SM, Greenberg D. Factors associated with off-label oncology prescriptions: the role of cost and financing in a universal healthcare system. Front Pharmacol. 2021;12:754390. https://doi.org/10.3389/fphar.2021.754390 34737706 PMC8560680

[61] Koleva-Kolarova R , Buchanan J, Vellekoop H, et al Financing and reimbursement models for personalised medicine: a systematic review to identify current models and future options. Appl Health Econ Health Policy. 2022;20:501-524. https://doi.org/10.1007/s40258-021-00714-9 35368231 PMC9206925

[62] Di Maio M , Perrone F, Conte P. Real-world evidence in oncology: opportunities and limitations. Oncologist. 2020;25:e746-e752. https://doi.org/10.1634/theoncologist.2019-0647 31872939 PMC7216461

