# Supplementary material for: Twenty-one-year report from the Danish Health Authority Expert Advisory Panel for review of treatment of 10 000 cancer patients
Source: Oncologist. 2025 May 8;30(5):oyaf059. doi: 10.1093/oncolo/oyaf059 (PMC12060716; doi:10.1093/oncolo/oyaf059)
Supplement: oyaf059_suppl_Supplementary_Appendix [file oyaf059_suppl_supplementary_appendix.docx]

**Supplementary Appendix**

**Links to annual reports from Danish Health Authority (DHA) (in Danish), 2003-2023.**

**2003:** [https://www.sst.dk/-/media/Udgivelser/2004/Publ2004/PLAN/Eksperimentel/Aarsrap2003_eksp_behl,-d-,pdf.ashx](https://eur03.safelinks.protection.outlook.com/?url=https%3A%2F%2Fwww.sst.dk%2F-%2Fmedia%2FUdgivelser%2F2004%2FPubl2004%2FPLAN%2FEksperimentel%2FAarsrap2003_eksp_behl%2C-d-%2Cpdf.ashx&data=05%7C02%7C%7C333d14ae54bc4cbfcb4808dca00a96a9%7C5968b90c51a64f088b4750ffffbe2e4f%7C0%7C0%7C638561217947130529%7CUnknown%7CTWFpbGZsb3d8eyJWIjoiMC4wLjAwMDAiLCJQIjoiV2luMzIiLCJBTiI6Ik1haWwiLCJXVCI6Mn0%3D%7C0%7C%7C%7C&sdata=zpu%2BgvdAhpIpcCxoXw5LQzGstoW9esCc00rfvvyyQZ4%3D&reserved=0)

**2004:** [https://www.sst.dk/-/media/Udgivelser/2005/Publ2005/PLAN/Eksperimentel/Aarsrap2004_eksp_behl,-d-,pdf.ashx](https://eur03.safelinks.protection.outlook.com/?url=https%3A%2F%2Fwww.sst.dk%2F-%2Fmedia%2FUdgivelser%2F2005%2FPubl2005%2FPLAN%2FEksperimentel%2FAarsrap2004_eksp_behl%2C-d-%2Cpdf.ashx&data=05%7C02%7C%7C333d14ae54bc4cbfcb4808dca00a96a9%7C5968b90c51a64f088b4750ffffbe2e4f%7C0%7C0%7C638561217947146542%7CUnknown%7CTWFpbGZsb3d8eyJWIjoiMC4wLjAwMDAiLCJQIjoiV2luMzIiLCJBTiI6Ik1haWwiLCJXVCI6Mn0%3D%7C0%7C%7C%7C&sdata=PkPuxMYGAkXXSAA%2BGBsk8NO8yA8fFW23JNah2tT%2FYY4%3D&reserved=0)

**2005:** [https://www.sst.dk/-/media/Udgivelser/2006/Publ2006/PLAN/Second_opinion/Secopinion_aars05,-d-,pdf.ashx](https://eur03.safelinks.protection.outlook.com/?url=https%3A%2F%2Fwww.sst.dk%2F-%2Fmedia%2FUdgivelser%2F2006%2FPubl2006%2FPLAN%2FSecond_opinion%2FSecopinion_aars05%2C-d-%2Cpdf.ashx&data=05%7C02%7C%7C333d14ae54bc4cbfcb4808dca00a96a9%7C5968b90c51a64f088b4750ffffbe2e4f%7C0%7C0%7C638561217947166939%7CUnknown%7CTWFpbGZsb3d8eyJWIjoiMC4wLjAwMDAiLCJQIjoiV2luMzIiLCJBTiI6Ik1haWwiLCJXVCI6Mn0%3D%7C0%7C%7C%7C&sdata=z0tEKdFSGsbygZPqYMlkKzydlDkN7po74EqP%2FILEfLs%3D&reserved=0)

**2006:** [https://www.sst.dk/-/media/Udgivelser/2007/Publ2007/PLAN/Eksperimentel/Aarsrap2006_eksp_behl,-d-,pdf.ashx](https://eur03.safelinks.protection.outlook.com/?url=https%3A%2F%2Fwww.sst.dk%2F-%2Fmedia%2FUdgivelser%2F2007%2FPubl2007%2FPLAN%2FEksperimentel%2FAarsrap2006_eksp_behl%2C-d-%2Cpdf.ashx&data=05%7C02%7C%7C333d14ae54bc4cbfcb4808dca00a96a9%7C5968b90c51a64f088b4750ffffbe2e4f%7C0%7C0%7C638561217947182300%7CUnknown%7CTWFpbGZsb3d8eyJWIjoiMC4wLjAwMDAiLCJQIjoiV2luMzIiLCJBTiI6Ik1haWwiLCJXVCI6Mn0%3D%7C0%7C%7C%7C&sdata=LFjCfDyZtU%2BWhpBMsR6%2BTKm6ZHq3jASqasNFFOY7z%2Fk%3D&reserved=0)

**2007:** [https://www.sst.dk/-/media/Udgivelser/2008/Publ2008/Plan/Eksperimentel_behandling/Aarsrap2007_eksperimentbehandling,-d-,pdf.ashx](https://eur03.safelinks.protection.outlook.com/?url=https%3A%2F%2Fwww.sst.dk%2F-%2Fmedia%2FUdgivelser%2F2008%2FPubl2008%2FPlan%2FEksperimentel_behandling%2FAarsrap2007_eksperimentbehandling%2C-d-%2Cpdf.ashx&data=05%7C02%7C%7C333d14ae54bc4cbfcb4808dca00a96a9%7C5968b90c51a64f088b4750ffffbe2e4f%7C0%7C0%7C638561217947192747%7CUnknown%7CTWFpbGZsb3d8eyJWIjoiMC4wLjAwMDAiLCJQIjoiV2luMzIiLCJBTiI6Ik1haWwiLCJXVCI6Mn0%3D%7C0%7C%7C%7C&sdata=xmVesb59GSZadmx6foyIpw5bWTy7pD66%2F3K1XqJ18cA%3D&reserved=0)

**2008**: [https://www.sst.dk/-/media/Udgivelser/2009/Publ2009/SUPL/Cancer/Eksperimentelbehl_aar2008,-d-,pdf.ashx](https://eur03.safelinks.protection.outlook.com/?url=https%3A%2F%2Fwww.sst.dk%2F-%2Fmedia%2FUdgivelser%2F2009%2FPubl2009%2FSUPL%2FCancer%2FEksperimentelbehl_aar2008%2C-d-%2Cpdf.ashx&data=05%7C02%7C%7C333d14ae54bc4cbfcb4808dca00a96a9%7C5968b90c51a64f088b4750ffffbe2e4f%7C0%7C0%7C638561217947200867%7CUnknown%7CTWFpbGZsb3d8eyJWIjoiMC4wLjAwMDAiLCJQIjoiV2luMzIiLCJBTiI6Ik1haWwiLCJXVCI6Mn0%3D%7C0%7C%7C%7C&sdata=zGApyTDvaTWq0ozfdcPE2gKXK2Qtf2%2FQxVWcOR9YjkU%3D&reserved=0)

**2009:** [https://www.sst.dk/-/media/Udgivelser/2010/Publ2010/PLAN/Eksperimentel/%C3%85rsrapport-2009-eksperimentel-behandling.ashx](https://eur03.safelinks.protection.outlook.com/?url=https%3A%2F%2Fwww.sst.dk%2F-%2Fmedia%2FUdgivelser%2F2010%2FPubl2010%2FPLAN%2FEksperimentel%2F%25C3%2585rsrapport-2009-eksperimentel-behandling.ashx&data=05%7C02%7C%7C333d14ae54bc4cbfcb4808dca00a96a9%7C5968b90c51a64f088b4750ffffbe2e4f%7C0%7C0%7C638561217947208206%7CUnknown%7CTWFpbGZsb3d8eyJWIjoiMC4wLjAwMDAiLCJQIjoiV2luMzIiLCJBTiI6Ik1haWwiLCJXVCI6Mn0%3D%7C0%7C%7C%7C&sdata=2D%2Btzn3vY24KTcFQD0czTn3WkBWA%2FyEbFN9%2Bh0hCaI4%3D&reserved=0)

**2010:** [https://www.sst.dk/-/media/Udgivelser/2011/Publ2011/SYB/EksperimentelBehandling/%C3%85rsrapport-2010-Eksperimentel-behandling.ashx](https://eur03.safelinks.protection.outlook.com/?url=https%3A%2F%2Fwww.sst.dk%2F-%2Fmedia%2FUdgivelser%2F2011%2FPubl2011%2FSYB%2FEksperimentelBehandling%2F%25C3%2585rsrapport-2010-Eksperimentel-behandling.ashx&data=05%7C02%7C%7C333d14ae54bc4cbfcb4808dca00a96a9%7C5968b90c51a64f088b4750ffffbe2e4f%7C0%7C0%7C638561217947214967%7CUnknown%7CTWFpbGZsb3d8eyJWIjoiMC4wLjAwMDAiLCJQIjoiV2luMzIiLCJBTiI6Ik1haWwiLCJXVCI6Mn0%3D%7C0%7C%7C%7C&sdata=GNvir4E5uc802xR%2FF61LJVWeutnfCzpuchzbnQB3Kmg%3D&reserved=0)

**2011:** [https://www.sst.dk/-/media/Udgivelser/2012/Publ2012/%C3%85rsrapport-2011,-d-,-Second-opinion-ordningen-og-eksperimentel-kr%C3%A6ftbehandling.ashx](https://eur03.safelinks.protection.outlook.com/?url=https%3A%2F%2Fwww.sst.dk%2F-%2Fmedia%2FUdgivelser%2F2012%2FPubl2012%2F%25C3%2585rsrapport-2011%2C-d-%2C-Second-opinion-ordningen-og-eksperimentel-kr%25C3%25A6ftbehandling.ashx&data=05%7C02%7C%7C333d14ae54bc4cbfcb4808dca00a96a9%7C5968b90c51a64f088b4750ffffbe2e4f%7C0%7C0%7C638561217947221406%7CUnknown%7CTWFpbGZsb3d8eyJWIjoiMC4wLjAwMDAiLCJQIjoiV2luMzIiLCJBTiI6Ik1haWwiLCJXVCI6Mn0%3D%7C0%7C%7C%7C&sdata=wOUMR%2Fk48%2Fwk%2BoYrv3Tk35yNd56hX1pVrpFYlpyQF8c%3D&reserved=0)

**2012:** [https://www.sst.dk/-/media/Udgivelser/2013/Second-opinion-ordningen-og-eksperimentel-kr%C3%A6ftbehandling.ashx](https://eur03.safelinks.protection.outlook.com/?url=https%3A%2F%2Fwww.sst.dk%2F-%2Fmedia%2FUdgivelser%2F2013%2FSecond-opinion-ordningen-og-eksperimentel-kr%25C3%25A6ftbehandling.ashx&data=05%7C02%7C%7C333d14ae54bc4cbfcb4808dca00a96a9%7C5968b90c51a64f088b4750ffffbe2e4f%7C0%7C0%7C638561217947228116%7CUnknown%7CTWFpbGZsb3d8eyJWIjoiMC4wLjAwMDAiLCJQIjoiV2luMzIiLCJBTiI6Ik1haWwiLCJXVCI6Mn0%3D%7C0%7C%7C%7C&sdata=31mNOGCBSW0tXMpIkUkJ%2Fb5%2BpPbDOO4tsGaq9e7bmVs%3D&reserved=0)

**2013**: [https://www.sst.dk/-/media/Udgivelser/2014/%C3%85rsrapport-2014-om-eksperimentiel-behandling-i-2013.ashx](https://eur03.safelinks.protection.outlook.com/?url=https%3A%2F%2Fwww.sst.dk%2F-%2Fmedia%2FUdgivelser%2F2014%2F%25C3%2585rsrapport-2014-om-eksperimentiel-behandling-i-2013.ashx&data=05%7C02%7C%7C333d14ae54bc4cbfcb4808dca00a96a9%7C5968b90c51a64f088b4750ffffbe2e4f%7C0%7C0%7C638561217947234646%7CUnknown%7CTWFpbGZsb3d8eyJWIjoiMC4wLjAwMDAiLCJQIjoiV2luMzIiLCJBTiI6Ik1haWwiLCJXVCI6Mn0%3D%7C0%7C%7C%7C&sdata=z805s6CQ42av2374vEVYALu9Vn6LoLRMrhgl2qOaQf4%3D&reserved=0)

**2014**: [https://www.sst.dk/-/media/Udgivelser/2015/R%C3%A5dgivning-vedr,-d-,-eksperimentiel-behandling-for-mennesker-med-livstruende-sygdomme.ashx](https://eur03.safelinks.protection.outlook.com/?url=https%3A%2F%2Fwww.sst.dk%2F-%2Fmedia%2FUdgivelser%2F2015%2FR%25C3%25A5dgivning-vedr%2C-d-%2C-eksperimentiel-behandling-for-mennesker-med-livstruende-sygdomme.ashx&data=05%7C02%7C%7C333d14ae54bc4cbfcb4808dca00a96a9%7C5968b90c51a64f088b4750ffffbe2e4f%7C0%7C0%7C638561217947240583%7CUnknown%7CTWFpbGZsb3d8eyJWIjoiMC4wLjAwMDAiLCJQIjoiV2luMzIiLCJBTiI6Ik1haWwiLCJXVCI6Mn0%3D%7C0%7C%7C%7C&sdata=VZNdhDBZO4OGufSHMFKXudFugptodzGsV%2BTiStuC4pE%3D&reserved=0)

**2015:** [https://www.sst.dk/-/media/Udgivelser/2016/R%C3%A5dgivning-om-eksperimentel-behandling-for-mennesker-med-livstruende-sygdom.ashx](https://eur03.safelinks.protection.outlook.com/?url=https%3A%2F%2Fwww.sst.dk%2F-%2Fmedia%2FUdgivelser%2F2016%2FR%25C3%25A5dgivning-om-eksperimentel-behandling-for-mennesker-med-livstruende-sygdom.ashx&data=05%7C02%7C%7C333d14ae54bc4cbfcb4808dca00a96a9%7C5968b90c51a64f088b4750ffffbe2e4f%7C0%7C0%7C638561217947246444%7CUnknown%7CTWFpbGZsb3d8eyJWIjoiMC4wLjAwMDAiLCJQIjoiV2luMzIiLCJBTiI6Ik1haWwiLCJXVCI6Mn0%3D%7C0%7C%7C%7C&sdata=vYBwbZh5Labhx%2Ff4wcLJlWn9Y9glmq6nQPlHre%2BRH5Q%3D&reserved=0)

**2016:** [https://www.sst.dk/-/media/Udgivelser/2017/R%C3%A5dgivning-om-eksperimentel-behandling-for-mennesker-med-livstruende-sygdom,-d-,-%C3%85rsrapport-2016.ashx](https://eur03.safelinks.protection.outlook.com/?url=https%3A%2F%2Fwww.sst.dk%2F-%2Fmedia%2FUdgivelser%2F2017%2FR%25C3%25A5dgivning-om-eksperimentel-behandling-for-mennesker-med-livstruende-sygdom%2C-d-%2C-%25C3%2585rsrapport-2016.ashx&data=05%7C02%7C%7C333d14ae54bc4cbfcb4808dca00a96a9%7C5968b90c51a64f088b4750ffffbe2e4f%7C0%7C0%7C638561217947252561%7CUnknown%7CTWFpbGZsb3d8eyJWIjoiMC4wLjAwMDAiLCJQIjoiV2luMzIiLCJBTiI6Ik1haWwiLCJXVCI6Mn0%3D%7C0%7C%7C%7C&sdata=rTYCVCny%2FBbOUMMoP3GDUgnlpH4GkAoiQ77A5bEgicU%3D&reserved=0)

**2017:** [https://www.sst.dk/-/media/Udgivelser/2018/%C3%85rsrapport---R%C3%A5dgivning-om-eksperimentel-behandling-for-mennesker-med-livstruende-sygdom.ashx](https://eur03.safelinks.protection.outlook.com/?url=https%3A%2F%2Fwww.sst.dk%2F-%2Fmedia%2FUdgivelser%2F2018%2F%25C3%2585rsrapport---R%25C3%25A5dgivning-om-eksperimentel-behandling-for-mennesker-med-livstruende-sygdom.ashx&data=05%7C02%7C%7C333d14ae54bc4cbfcb4808dca00a96a9%7C5968b90c51a64f088b4750ffffbe2e4f%7C0%7C0%7C638561217947258589%7CUnknown%7CTWFpbGZsb3d8eyJWIjoiMC4wLjAwMDAiLCJQIjoiV2luMzIiLCJBTiI6Ik1haWwiLCJXVCI6Mn0%3D%7C0%7C%7C%7C&sdata=F5h5jpugihuwZrXV%2BKmR%2F%2FZwYm6YwlIYBD4PFVtgtJU%3D&reserved=0)

**2018:** [https://www.sst.dk/-/media/Udgivelser/2019/R%C3%A5dgivning-om-eksperimentel-behandling-2018.ashx](https://eur03.safelinks.protection.outlook.com/?url=https%3A%2F%2Fwww.sst.dk%2F-%2Fmedia%2FUdgivelser%2F2019%2FR%25C3%25A5dgivning-om-eksperimentel-behandling-2018.ashx&data=05%7C02%7C%7C333d14ae54bc4cbfcb4808dca00a96a9%7C5968b90c51a64f088b4750ffffbe2e4f%7C0%7C0%7C638561217947264449%7CUnknown%7CTWFpbGZsb3d8eyJWIjoiMC4wLjAwMDAiLCJQIjoiV2luMzIiLCJBTiI6Ik1haWwiLCJXVCI6Mn0%3D%7C0%7C%7C%7C&sdata=RYNv8c3PjUCNdznAJbbU2%2FQck0aUWumiITni%2BjK4W7g%3D&reserved=0)

**2019:** [https://www.sst.dk/-/media/Udgivelser/2020/Aarsrapport-2019-eksperimentel-behandling.ashx](https://eur03.safelinks.protection.outlook.com/?url=https%3A%2F%2Fwww.sst.dk%2F-%2Fmedia%2FUdgivelser%2F2020%2FAarsrapport-2019-eksperimentel-behandling.ashx&data=05%7C02%7C%7C333d14ae54bc4cbfcb4808dca00a96a9%7C5968b90c51a64f088b4750ffffbe2e4f%7C0%7C0%7C638561217947270024%7CUnknown%7CTWFpbGZsb3d8eyJWIjoiMC4wLjAwMDAiLCJQIjoiV2luMzIiLCJBTiI6Ik1haWwiLCJXVCI6Mn0%3D%7C0%7C%7C%7C&sdata=NwVpB4UBNlXRTU4ZCnCleukOxe68MRFrQcgmLqkLufY%3D&reserved=0)

**2020:** [Rådgivning om eksperimentel behandling - for mennesker med livstruende sygdom (sst.dk)](https://www.sst.dk/-/media/Udgivelser/2021/Eksperimentel-behandling/AArsrapport-2020-Eksperimentel-behandling.ashx)

**2021**: [https://www.sst.dk/-/media/Udgivelser/2022/Eksperimentel-behandling/AArsrapport-eksperimentel-behandling-2021.ashx](https://eur03.safelinks.protection.outlook.com/?url=https%3A%2F%2Fwww.sst.dk%2F-%2Fmedia%2FUdgivelser%2F2022%2FEksperimentel-behandling%2FAArsrapport-eksperimentel-behandling-2021.ashx&data=05%7C02%7C%7C333d14ae54bc4cbfcb4808dca00a96a9%7C5968b90c51a64f088b4750ffffbe2e4f%7C0%7C0%7C638561217947275822%7CUnknown%7CTWFpbGZsb3d8eyJWIjoiMC4wLjAwMDAiLCJQIjoiV2luMzIiLCJBTiI6Ik1haWwiLCJXVCI6Mn0%3D%7C0%7C%7C%7C&sdata=5tCJrNHfsLow6W16hKke%2B7o7a4vIZdU9wkQfQWBWaV0%3D&reserved=0)

**2022** [https://www.sst.dk/-/media/Udgivelser/2023/Eksperimentel-behandling/Aarsrapport-Sundhedsstyrelsens-raadgivende-panel-2022.ashx](https://eur03.safelinks.protection.outlook.com/?url=https%3A%2F%2Fwww.sst.dk%2F-%2Fmedia%2FUdgivelser%2F2023%2FEksperimentel-behandling%2FAArsrapport-Sundhedsstyrelsens-raadgivende-panel-2022.ashx&data=05%7C02%7C%7C333d14ae54bc4cbfcb4808dca00a96a9%7C5968b90c51a64f088b4750ffffbe2e4f%7C0%7C0%7C638561217947281739%7CUnknown%7CTWFpbGZsb3d8eyJWIjoiMC4wLjAwMDAiLCJQIjoiV2luMzIiLCJBTiI6Ik1haWwiLCJXVCI6Mn0%3D%7C0%7C%7C%7C&sdata=LB7P%2Ba4nnQUSIhbnE8L3BIt%2FMCcsN%2F3%2BIDO1RcIJ0Bg%3D&reserved=0)

**2023** https://www.sst.dk/da/udgivelser/2024/Raadgivning-om-eksperimentel-behandling-af-mennesker-med-livstruende-sygdom-Aarsrapport-2023

**Links to relevant laws and announcements regulating the Danish Health act in 2003-2023 (in Danish).**

- Retsinformation. Bekendtgørelse om ret til sygehusbehandling m.v. BEK nr 958 af 29/08/2014. Published 2014. <https://www.retsinformation.dk/eli/lta/2014/958>
- Retsinformation. Bekendtgørelse om ret til sygehusbehandling m.v. BEK nr 657 af 28/06/2019. Published 2019. <https://www.retsinformation.dk/eli/lta/2019/657>
- Sundheds-ældreministeriet. Princippapir om prioritering for sygehuslægemidler. Published 2016. <https://www.regioner.dk/media/4119/folketingets-7-principper-for-prioritering-af-sygehuslaegemidler.pdf>
