# Supplementary material for: Twenty-one-year report from the Danish Health Authority Expert Advisory Panel for review of treatment of 10 000 cancer patients
Source: Oncologist. 2025 May 8;30(5):oyaf059. doi: 10.1093/oncolo/oyaf059 (PMC12060716; doi:10.1093/oncolo/oyaf059)
Supplement: oyaf059_suppl_Supplementary_Tables_S1_Figures_S1-S2 [file oyaf059_suppl_supplementary_tables_s1_figures_s1-s2.docx]

**Supplementary Table S1. Causes of administrative rejection of requests***

|  | **Number of cases** | **Pct.** |
| --- | --- | --- |
| Suggested treatment available as SoC | 66 | 53% |
| Second opinion on established treatment | 23 | 18% |
| Suggested treatment already started or planned | 14 | 11% |
| Request referred to a national center | 9 | 7% |
| Patient not eligble for treatment | 8 | 6% |
| Not a life-threatening condition | <5 | NA |
| Other causes | <5 | NA |
| **Total** | **125** | **100%** |

*Registered only for 2018 and 2021-23.

**Supplementary Figure S1. The relative distribution of tumor types or primary sites of evaluated cases per year of referral (2003-23).**

Color codes according to primary tumor site or type: Green-blue, neuroendcrine tumors; light brown, malignant melanomas; dark gray, rare tumors/tumors of multiple or unknown sites; dark brown, CNS tumors; dark blue: breast tumors; green, head-and-neck tumors; light blue, urinary tract and prostatic tumors; yellow, gynecological tumors; gray, lung and pleural tumors; orange, upper gastro-intestinal, pancreatic and liver tumors; blue: colo-rectal tumors.

**Supplementary Figure S2. Relative distribution of categories of primary advice given by the Expert Advisory Panel per year of referral (2003-2023).**

Color codes according to type of primary advise: Green, no further suggestions; light blue, suggested treatment endorsed; yellow, advise on further standard of care treatment, further investigations, or 2^nd^ opinion of treatment; gray, advise on treatment in a clinical trial; orange, non-reimbursed treatment recommended; dark blue, treatment abroad recommended.
